# Spatial Mechanisms for Segregation of Competing Sounds, and a Breakdown in Spatial Hearing

**DOI:** 10.3389/fnins.2020.571095

**Published:** 2020-09-16

**Authors:** John C. Middlebrooks, Michael F. Waters

**Affiliations:** ^1^Departments of Otolaryngology, Neurobiology and Behavior, Cognitive Sciences, and Biomedical Engineering, University of California, Irvine, Irvine, CA, United States; ^2^Department of Neurology, Barrow Neurological Institute, Phoenix, AZ, United States

**Keywords:** spatial release from masking, interaural time difference (ITD), interaural level difference (ILD), cat, rhythmic masking release, cerebellar ataxia, Kv3.3

## Abstract

We live in complex auditory environments, in which we are confronted with multiple competing sounds, including the cacophony of talkers in busy markets, classrooms, offices, etc. The purpose of this article is to synthesize observations from a series of experiments that focused on how spatial hearing might aid in disentangling interleaved sequences of sounds. The experiments were unified by a non-verbal task, “rhythmic masking release”, which was applied to psychophysical studies in humans and cats and to cortical physiology in anesthetized cats. Human and feline listeners could segregate competing sequences of sounds from sources that were separated by as little as ∼10°. Similarly, single neurons in the cat primary auditory cortex tended to synchronize selectively to sound sequences from one of two competing sources, again with spatial resolution of ∼10°. The spatial resolution of spatial stream segregation varied widely depending on the binaural and monaural acoustical cues that were available in various experimental conditions. This is in contrast to a measure of basic sound-source localization, the minimum audible angle, which showed largely constant acuity across those conditions. The differential utilization of acoustical cues suggests that the central spatial mechanisms for stream segregation differ from those for sound localization. The highest-acuity spatial stream segregation was derived from interaural time and level differences. Brainstem processing of those cues is thought to rely heavily on normal function of a voltage-gated potassium channel, Kv3.3. A family was studied having a dominant negative mutation in the gene for that channel. Affected family members exhibited severe loss of sensitivity for interaural time and level differences, which almost certainly would degrade their ability to segregate competing sounds in real-world auditory scenes.

## Introduction

Everyday listening situations require us to isolate sounds of interest amid competing sounds. The classic example is the “cocktail party problem” (Cherry, 1953), but more quotidian examples include busy offices, classrooms, restaurants, etc. Spatial hearing has long been thought to aid in sorting out these complex auditory scenes. For instance, Cherry listed “the voices come from different directions” as a likely factor in recognizing what one person is saying when others are speaking (Cherry, 1953). *Spatial release from masking* refers to the condition in which detection or recognition of a sound of interest, the *target*, is enhanced when the target source is separated in space from sources of competing sounds, the *maskers* (Hirsh, 1950; Zurek, 1993; Kidd et al., 1998).

Spatial hearing can be especially beneficial in the task of *stream segregation* (Shinn-Cunningham, 2005; Marrone et al., 2008). Stream segregation refers to the ability to sort temporally interleaved sequences of sounds into distinct perceptual streams. Cherry’s early study can be regarded as an example of spatial stream segregation (SSS), in which two competing speech streams were more intelligible when presented through separate headphones than when the two streams were mixed and the combined sounds presented to one or both headphones (Cherry, 1953). More recent reports have argued that spatial cues are weaker segregation cues than are fundamental frequency or spectral envelope (reviewed by Moore and Gockel, 2002). Weak spatial effects, however, are most often found in studies of *obligatory* stream segregation in which performance of a psychophysical task requires a listener to fuse information across two or more streams that might be segregated by spatial or other cues (Boehnke and Phillips, 2005; Stainsby et al., 2011; Füllgrabe and Moore, 2012).

Robust spatial effects on stream segregation are observed in studies of *voluntary* stream segregation in which the listener must evaluate a single stream in the presence of competing sounds (e.g., Hartmann and Johnson, 1991; Ihlefeld and Shinn-Cunningham, 2008). In particular, SSS is important for tasks that require a listener to piece together the successive syllables from one talker while excluding sounds from other talkers (Shinn-Cunningham, 2005; Kidd et al., 2008; Marrone et al., 2008). That is the task of a listener in a real-world cocktail party, and it applies to many other everyday listening situations.

The purpose of this review is to synthesize observations from a series of reported experiments that isolated spatial attributes of stream segregation. We address the question: “What is going on in the brain under conditions in which competing sound sequences are heard as segregated”? We review psychophysical and physiological experiments in humans and cats, and we review a natural experiment in which sensitivity to fundamental cues for SSS was lost due to a gene mutation. The results of those studies suggest that the multiple auditory objects in a cocktail party or other complex auditory scene activate multiple distinct ensembles of neurons in a listener’s auditory cortex, each ensemble synchronized to a particular auditory source.

## Spatial Stream Segregation in Humans and in an Animal Model

Psychophysical studies of spatial stream segregation have been conducted using human and feline listeners (Middlebrooks and Onsan, 2012; Javier et al., 2016). Experiments with normal-hearing human listeners are important because of the importance of SSS in solving everyday human hearing challenges. The use of an animal model has enabled parallel psychophysical and invasive physiological studies.

“Rhythmic masking release” was originally devised as a psychophysical test of stream segregation using headphone-presented dichotic cues (Sach and Bailey, 2004). Middlebrooks and Onsan (2012) adapted that task for the free field, isolating spatial contributions to stream segregation while eliminating pitch, spectral, and other putative streaming cues. Target and masker sequences were constructed of temporally interleaved sequences of noise bursts having identical long-term spectra but no temporal overlap. Humans and cats were required to discriminate between rhythms of target sequences in the presence of interleaved masker sequences presented from varying source locations. Success in discriminating the rhythms required perceptual segregation of target and masker streams.

**FIGURE 1:**
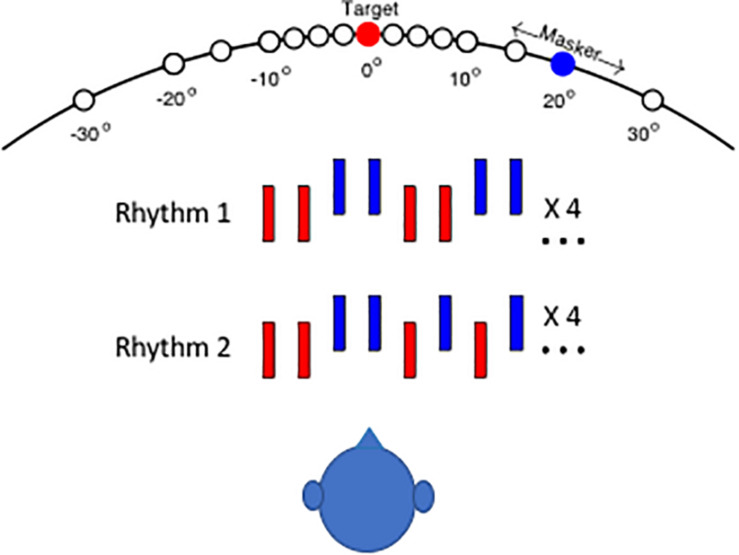
Schematic of testing configuration and stimulus rhythms. The listener was seated in an anechoic chamber in the center of a horizontal array of small loudspeakers that were positioned at 5 or 10° intervals. The target source (denoted by red) was fixed at 0°, and the masker source location (blue) was varied parametrically in azimuth (i.e., the horizontal dimension). In the 40° target condition, the listener was turned to face 40° left, placing the target 40° to his/her right. Stimulus Rhythm 1 or 2 consisted of a sequence of noise bursts (denoted by red bars) that were interleaved with masker noise bursts (blue). The vertical offset of blue bars in the illustration indicates that target and masker sources could differ in azimuth.

The rhythmic patterns and the layout of stimulus sources for the human psychophysical task are shown schematically in Figure 1. In the depictions of the two rhythms, red and blue bars denote noise bursts from the target and masker sequences, respectively. In the illustration, the vertical offset of signal and masker sound bursts denotes a difference in horizontal source location. On each trial, the listener was required to report whether he or she heard Rhythm 1 or Rhythm 2. On trials in which signal and masker sources were separated sufficiently, the target rhythm tended to pop out from the masker, and the rhythm was clearly recognizable. In an animal version of the task, cats pressed a pedal to begin presentation of Rhythm 1. When they detected a change to Rhythm 2, they could release the pedal to receive a food reward (Javier et al., 2016).

**FIGURE 2:**
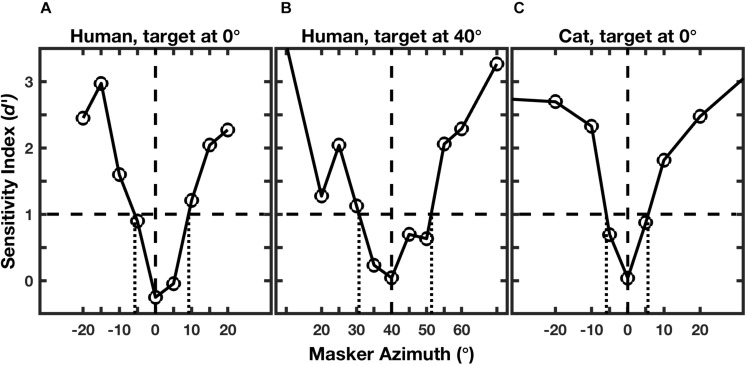
Rhythmic masking release (RMR). The curves represent performance at rhythm identification as a function of masker location. Performance is given by the sensitivity index (*d’*), which is an unbiased measure that incorporates rates of correct and incorrect identifications (Green and Swets, 1966; MacMillan and Creelman, 2005). The horizontal dashed line indicates the threshold criterion of *d’* = 1, and the vertical dotted lines indicate the projections of threshold crossings to the masker-azimuth axis, giving the RMR thresholds. The three panels represent: **(A)** an individual human listener; **(B)** the same human listener turned to place the target 40° to the right; and **(C)** an individual feline listener with the target straight ahead. (From Middlebrooks and Onsan, 2012; and Javier et al., 2016).

Figure 2 shows examples of performance of an individual human for targets located at 0° and 40° (Figures 2A,B; Middlebrooks and Onsan, 2012) and for an individual cat for a target at 0° (Figure 2C; Javier et al., 2016). Performance for each masker location was given by the sensitivity index, *d’*, where values of *d’* around zero indicate random-chance performance, and values ≥1 were taken as above threshold (Green and Swets, 1966; MacMillan and Creelman, 2005). As expected, the human and feline listeners were unable to recognize the rhythms when the masker locations were close to the target locations of 0° (Figures 2A,C) or 40° (Figure 2B). For both species, however, performance improved markedly as the target-masker separation was increased to about 10° or greater.

**FIGURE 3:**
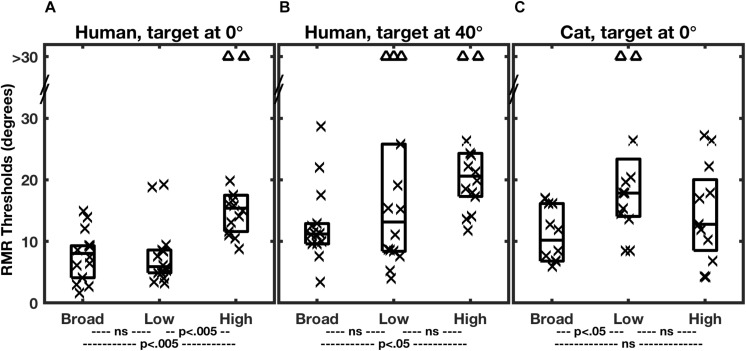
Summary of RMR thresholds. In each panel, each column of a box and symbols represents the distribution of thresholds among 7 human listeners **(A,B)** or 6 feline listeners **(C)** in one passband condition. Thresholds for masker sources to the left and right of the target were combined, so that each listener is represented by two symbols. Symbols indicate individual thresholds, and boxes indicate 25th, 50th, and 75th percentiles. The triangles near the top of each panel denote instances in which threshold performance was not attained for a maximum target/masker separation of 30°. Broad, low, and high indicate the broadband, low-band, and high-band passband conditions described in the text. In each species/target location condition, thresholds varied significantly with passband. The statistical *p* values across the bottom of each panel indicate results of *post hoc* pair-wise comparisons of passband conditions with Bonferroni correction. (From Middlebrooks and Onsan, 2012; and Javier et al., 2016).

Figure 3 shows the distributions of RMR thresholds of human and feline listeners from the two studies in various stimulus-passband and target-location conditions. Individual thresholds are denoted by symbols, and the boxes represent medians and 25th and 75th quartiles for each condition. The broad-band stimulus condition is represented by the left-most column of each panel. Notably, broadband SSS by human and cat listeners in the two studies was comparable in acuity. The median RMR thresholds in the broad-band condition with the target at 0° were 8.1° for the human listeners and 10.2° for the cats. The similarity in psychophysical results between humans and cats, at least in the broad-band condition, adds validity to the cat as a model for humans in invasive physiological studies. Differences between the species appeared when restricted stimulus passbands were tested, considered in the next section.

Performance for the human listeners was somewhat degraded when the target was displaced 40° to the side (Figure 3B; Middlebrooks and Onsan, 2012). The median RMR threshold in the broad-band condition increased from 8.1° for the straight-ahead, 0°, target to 11.2° for the target at 40°. That the threshold separations were wider for the lateral target is not surprising, given that the spatial rate of change of interaural difference cues tends to decline with increasing distance from the midline (Shaw, 1974; Kuhn, 1977). What is notable is that performance was not very much worse. A popular model of spatial representation in the auditory cortex has it that the location of a given stimulus is represented by the balance of activity between broadly tuned “opponent” neural populations tuned to the right or left half of space (Stecker et al., 2005; Phillips, 2008; Magezi and Krumbholz, 2010; Briley et al., 2013). In the measure of SSS in the 40°-target condition, however, the target and all of the masker location were restricted to the right hemifield of space, meaning that all the stimuli were primarily activating neurons in the left cortical hemisphere. That raises the possibility that listeners performed SSS primarily on the basis of computations within one cortical hemisphere, that is, with little or no inter-hemisphere comparison. That speculation is further supported by single-neuron recordings in cats (Middlebrooks and Bremen, 2013), presented in a later section.

This section has reviewed psychophysical experiments that demonstrated a robust spatial contribution to stream segregation, both in humans and cats. Relevant to the example of a cocktail party, the minimum spatial resolution of SSS reported for humans was somewhat narrower than the width of a human head at arm’s length. Compared to a condition in which target and maskers were located around the frontal midline, human listeners showed only minor degradation of performance when all stimulus and masker source were restricted to one half of space. We now turn to the spatial acoustical cues that underlie SSS, which further inform notions of brain mechanism of SSS.

## Spatial Cues for Stream Segregation

The locations of sound sources are not represented directly in the auditory periphery but must be inferred from spatial cues provided by the interaction of incident sounds with the acoustics of the head and external ears. The principal spatial cues in the horizontal dimension are interaural differences in the timing of cycle-by-cycle fine structure (ITD_fs_) and interaural differences in sound pressure level (ILD), reviewed by Middlebrooks and Green (1991). Other possible spatial cues include interaural differences in the timing of sound envelopes (ITD_env_), a monaural level sensitivity referred to as the “head-shadow effect”, and spectral shape cues. The utility of various cues for spatial hearing varies with sound frequency, with ITD_fs_ cues being audible by humans only below ∼1.4 kHz (Brughera et al., 2013), and ILD cues generally increasing in magnitude with frequency increasing above 4 kHz. Identification of the spatial cues that support SSS has raised important insights into the brain mechanisms for SSS as well as providing some practical guidance for remediation of hearing impairment.

Middlebrooks and Onsan (2012) evaluated SSS performance by human listeners using stimuli that differentially favored ITD_fs_ or ILD cues; the control condition was SSS performance with a broadband stimulus, 400 to 16000 Hz in passband. Results from that study are shown in Figures 3A,B. The low-band stimulus, 400 to 1600 Hz, essentially eliminated ILD cues, leaving ITD_fs_ as the principal spatial cue in the horizontal dimension. In that condition, SSS performance was not significantly different from that in the control, broadband, condition. In contrast, the spatial acuity of SSS was markedly degraded in the high-band condition, which eliminated ITD_fs_ cues, leaving only ILD cues. Middlebrooks and Onsan (2012) interpreted those observations to mean that humans receive their highest-acuity spatial cues for SSS from ITD_fs_ cues.

A different result was obtained for cats by Javier and colleagues (2016; Figure 3C). The cats consistently showed degraded performance in the low-band condition (i.e., using ITD_fs_ cues) and control-level performance in the high-band condition, presumably using ILD cues. Those results were taken to indicate that cats receive their highest-acuity SSS cues from ILDs. Javier et al. (2016) suggested that the difference between humans and cats in use of ITD_fs_ and ILD cues could be accounted for in large part by differences in the sizes of the heads of the two species (Javier et al., 2016).

An additional interaural difference cue to consider is the interaural time difference in the envelopes of high-frequency sounds (ITD_env_). In humans, Middlebrooks and Onsan (2012) evaluated stream segregation in high-frequency sounds (4000 to 16000 Hz) presented over headphones, manipulating ILD and ITD independently. The results of those experiments showed that high-frequency spatial stream segregation relies almost entirely on ILD cues, with only a slight synergy with ITD_env_ and only at the largest physiologically relevant ITDs, around 700 μs.

Studies of spatial release from masking have emphasized the importance of the monaural *head-shadow effect* (Bronkhorst and Plomp, 1988; Hawley et al., 2004). When a target and a masker are separated in space, shadowing by the head will result in a difference in the target-to-masker ratio at the two ears. In the RMR task, the head shadow could produce a systematic fluctuation between target and masker sound levels at each ear. Middlebrooks and Onsan (2012) tested conditions in which the sound sequences varied randomly in sound level, thereby confounding any monaural level cues. The variable-level stimuli produced essentially no degradation in stream segregation, suggesting that the principal spatial cues are interaural difference cues (ITD_fs_ and/or ILD), which would not be confounded by the level variation.

The observation that normal-hearing listeners derive their highest-acuity SSS from ITD_fs_ cues (Middlebrooks and Onsan, 2012) is significant for the remediation of hearing impairment with hearing aids or cochlear implants. In general, hearing aids and cochlear implants do a poor job of transmitting ITD_fs_ information. Hearing aids introduce delays of as great as 10000 μs, and those delays can vary substantially across frequencies (e.g., Dillon et al., 2003). A device-imposed delay of, say, 7000 μs is about an order of magnitude larger than the maximum naturally occurring ITD_fs_. Cochlear implant sound processors, on the other hand, transmit only the envelopes of sounds, eliminating temporal fine structure altogether. Moreover, when tested with laboratory processors, implant users show only limited sensitivity to temporal fine structure (e.g., Zeng, 2002; van Hoesel, 2007). The demonstration of the importance of ITD_fs_ cues for SSS should heighten the motivation for overcoming those failings in delivering temporal fine structure to hearing aid and cochlear implant users.

**FIGURE 4:**
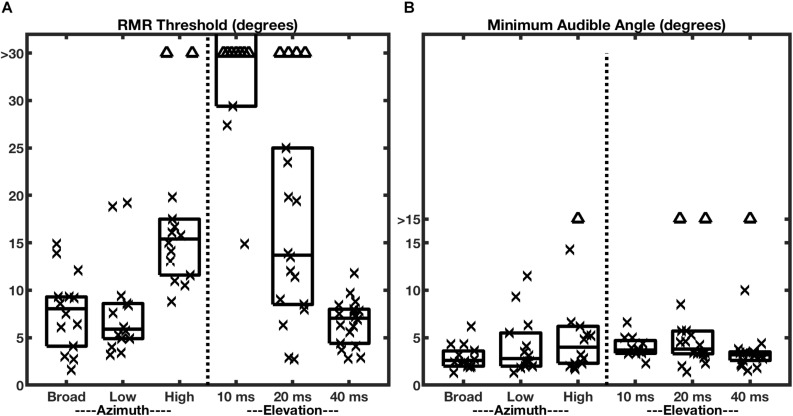
RMR thresholds vary more across bandwidth and burst durations more than do MAAs. The two panels represent RMR thresholds **(A)** and MAAs **(B)** for human listeners. Each column of a box and symbols represents one passband condition in the horizontal (azimuth) dimension (burst duration 20 ms) or one burst-duration condition in the vertical midline (elevation); box and symbol conventions are as in Figure 3. The left halves of the panels show data for various passbands tested in the horizontal plane with the target at 0° azimuth. The right halves show data for various broadband burst durations tested in the vertical midline with the target at 0° elevation. (From Middlebrooks and Onsan, 2012).

Given the results reviewed so far, one might question whether SSS should be regarded as a truly spatial phenomenon, or whether it merely reflects stream segregation on the basis of interaural differences. Middlebrooks and Onsan (2012) addressed that issue by presenting target and masker sources in the vertical midline. In that condition, interaural differences are negligible, and the principal spatial cues are spectral-shape cues provided by the elevation-specific filtering properties of the external ears (reviewed by Middlebrooks and Green, 1991). Those experiments demonstrated that spatial stream segregation is possible in elevation, i.e., in the absence of interaural difference cues. Nevertheless, they also revealed an unexpected dependence on the durations of the individual stimulus noise bursts that constituted the stimulus sequences (Figure 4A, right half of the panel). When the noise bursts were shortened to 10 ms in duration, the RMR task was impossible for most of the listeners, whereas that duration produced essentially no decline in horizontal resolution. When the burst duration was lengthened to 40 ms, however, stream segregation in elevation improved markedly, so that the median RMR threshold in elevation was not significantly different from that in azimuth. Those results indicate that SSS is not strictly an interaural-difference phenomenon. Nevertheless, they show that the mechanisms for deriving cues for SSS from spectral shapes appear to require greater temporal integration than do those for processing interaural cues.

The minimum audible angle (MAA) is a measure of the spatial acuity of sound-source localization. Middlebrooks and Onsan (2012) measured MAAs in the same human listeners that were tested for SSS; those MAA data are shown in Figure 4B. In the broadband, azimuth, condition (left-most box and symbols in Figure 4, panels A and B), nearly all the RMR thresholds were wider than the MAAs, although the distributions were contiguous. The most remarkable observation about the MAAs, however, is that the median values of MAAs in azimuth were largely constant across varying passbands and, in the vertical midline, were largely constant across burst durations. This contrasts with RMR thresholds (Figure 4A), which varied markedly across those stimulus conditions.

One might have entertained the hypothesis that static location discrimination (i.e., measured by MAA) and SSS draw spatial information from a common cortical spatial representation. That hypothesis, however, would predict that localization and SSS would show similar trends in spatial acuity across passband and burst-duration conditions. The results shown in Figure 4 clearly refute that prediction. Based on those human psychophysical results, Middlebrooks and Onsan (2012) raised the possibility that SSS is derived from different cortical mechanisms than those that underlie sound-source localization. Location discrimination and SSS almost certainly rely on common mechanisms for low-level analysis of ITD_fs_, ILD, and spectral shape. At more central levels, however, SSS appears to derive highest horizontal acuity from ITD_fs_ cues and to require greater temporal integration for use of spectral-shape cues for the vertical dimension.

Several additional lines of evidence support the view that the mechanisms that underlie SSS (or spatial release from masking) are distinct from those for source localization. First, neural recordings in anesthetized cats have demonstrated largely similar spatial sensitivity among several primary auditory cortical areas (Harrington et al., 2008). Nevertheless, reversible inactivation of a subset of those areas disrupts performance of a localization task (Malhotra et al., 2004), whereas inactivation of another area disrupts performance of a rhythm-discrimination task while preserving localization (Lomber and Malhotra, 2008). Second, a speech study demonstrated essentially equivalent spatial unmasking of speech by ITD and ILD cues across conditions that produced markedly different spatial percepts (Edmonds and Culling, 2005). Finally, a population of patients having a variety of cortical lesions displayed a dissociation between those who showed deficits in a lateralization task and others who showed impaired spatial release from masking (Duffour-Nikolov et al., 2012).

## Spatial Stream Segregation in the Ascending Auditory Pathway

We now return to the question: “What is going on in the brain under stimulus conditions in which a listener could segregate interleaved sound sequences”? We consider two contrasting hypotheses. One is that the spatial relations of sound sources in the auditory scene are faithfully transmitted to early stages of the auditory cortex and that “higher” cortical mechanisms in some way segregate sounds based on that low-level cortical representation. The other view is that the job of spatial stream segregation is carried out by the auditory brainstem and that segregated streams are represented in the auditory cortex as distinct populations of activated neurons. Middlebrooks and Bremen (2013) tested those hypotheses by recording from single neurons in the primary auditory cortex (area A1) of anesthetized cats. The rationale was that higher-level cortical mechanisms are largely suppressed under anesthesia. For that reason, the first hypothesis, which demands higher-order cortical processing, would predict little or no spatial stream segregation in the anesthetized cortex. Conversely, the second hypothesis, which calls for stream segregation in the auditory brainstem, would predict that spatial stream segregation would be evident in the cortex under anesthesia.

**FIGURE 5:**
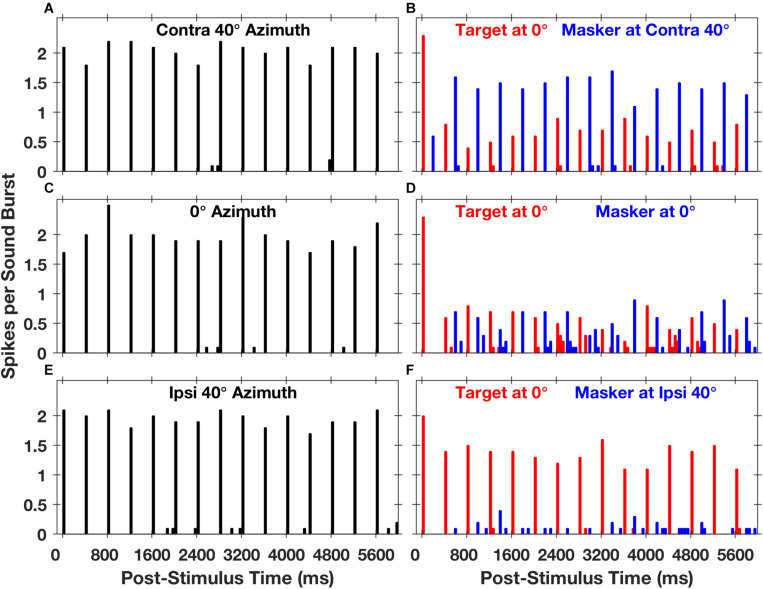
Spike counts from an isolated single neuron synchronized to target and masker sound sequences. The plots are post-stimulus-time histograms of spikes synchronized to sequences of 5-ms sound bursts from a single source **(A,C,E)** at a rate of 2.5 bursts per second or to interleaved sequences from two sources **(B,D,F)** at an aggregate rate of 5 bursts per second. The histogram bars represent mean spike counts in 50-ms time bins, averaged over 20 repetitions. In the left column of panels, the sound source was located in the horizontal plane at contralateral 40° azimuth **(A)**, 0° azimuth **(C)**, or ipsilateral 40° azimuth **(E)**; contralateral and ipsilateral are with respect to the recording site in the right cortical hemisphere. In the right column of panels, red or blue bars represent spikes synchronized to the target or the masker, respectively. The target source was fixed in location at 0°, and the masker source was located at contralateral 40° **(B)**, 0° **(D)**, or ipsilateral 40° **(F)**. The condition in panel **(D)**, in which target and masker were co-located at 0°, is identical to the condition in panel **(C)** except that the sound-burst rate is doubled. Unit 1204.3.10. (From Middlebrooks and Bremen, 2013).

Stimuli in the Middlebrooks and Bremen study consisted of trains of broad-band noise bursts presented from target and masker sources located in the horizontal plane, much as in the cat psychophysical experiments (Javier et al., 2016). Extracellular spikes recorded from cortical neurons tended to synchronize closely with the stimulus noise bursts. Figure 5 shows post-stimulus-time histograms representing the responses of one well-isolated single neuron (Middlebrooks and Bremen, 2013). The left panels (Figures 5A,C,E) show the responses to sounds from a single source located straight ahead (0°) or at 40⋅contralateral or ipsilateral to the midline. Spike times were largely restricted to the 50-ms-wide time bins following the onsets of noise bursts. The spike rates of this neuron elicited by a train of noise burst from a single source showed essentially no sensitivity to the locations of sources across the 80° range shown in the illustration, as indicated by the similar heights of bars in panels 5A, C, and E.

The spatial sensitivity of neurons was substantially increased in the presence of a competing sound. The right panels in Figure 5 show responses synchronized to a target fixed at 0° (denoted by red bars) and a masker (blue bars) presented from contralateral 40°, 0°, or ipsilateral 40°. In each condition, there was a robust response to the first noise burst in the sequence (at 0 ms), but the response to the second noise burst, at 200 ms, was weak or entirely suppressed. In the condition shown in Figure 5D, the target and masker were co-located at 0°; this is an identical condition to that shown in Figure 5C except that the rate of presentation of the noise bursts was doubled. At this higher presentation rate, the response to each burst was less than half of that at the slower rate, and the precision of synchrony was somewhat degraded. When the masker source was moved to ipsilateral 40°, however, there was a striking recovery of the response to the target and nearly complete suppression of the response to the masker (Figure 5F). Conversely, when the masker source was moved to contralateral 40°, the neural response was largely captured by the masker, with corresponding suppression of the response to the target (Figure 5B). Middlebrooks and Bremen (2013) took this pattern of responses as evidence for SSS in the responses of a single cortical neuron.

**FIGURE 6:**
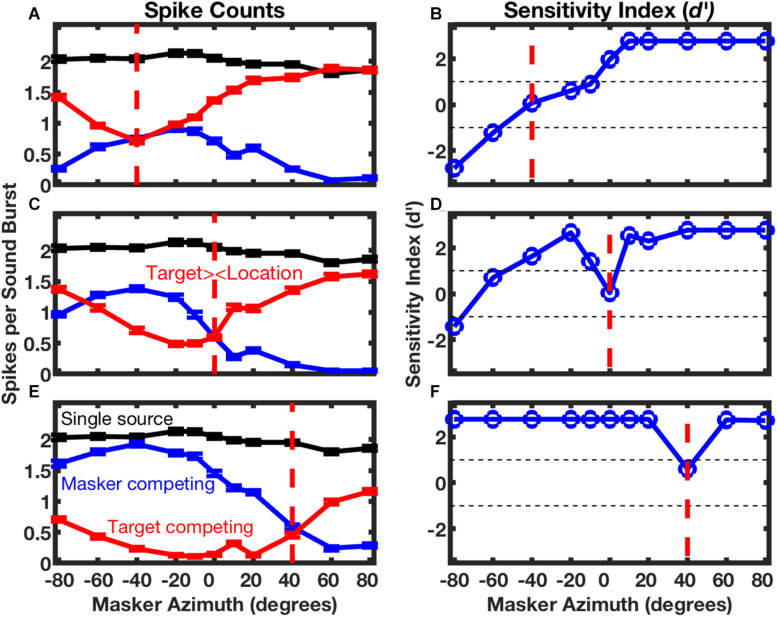
Spatial stream segregation in the responses of a single neuron. Responses of the neuron represented in Figure 5 are plotted here as a function of masker azimuth. The location of the target source was fixed at contralateral 40° **(A,B)**, 0° **(C,D)**, or ipsilateral 40° **(E,F)**, as indicated in each panel by a vertical dashed line. The left column of panels shows mean spike counts per sound burst synchronized to a single source (black, duplicated in each panel), or to competing target (red) or masker (blue) sources. Error bars are standard errors of the mean. The right column of panels plots the sensitivity index (*d’*) for discrimination of trial-by-trial mean spike rates synchronized to the target or masker. Positive values of *d’* denote cases in which there were more spikes synchronized to the more contralateral source. Unit 1204.3.10. (From Middlebrooks and Bremen, 2013).

The responses of the neuron in Figure 5 are shown in finer spatial detail in Figure 6; the three panels on the left of Figure 6 show stimulus-synchronized spike counts measured for three target locations, with the target location for each panel denoted by the vertical dashed line. The blue lines represent counts of spikes that were synchronized to the masker as a function of masker location. The red lines represent counts synchronized to the fixed-location target indicated by the vertical red dashed line; those responses also varied as a function of masker location. The black lines, duplicated in each of the panels, represent spike counts synchronized to a single source. When the target and masker sources were co-localized (i.e., when the blue line crossed the vertical dashed line), the target and masker spike counts were essentially identical, and both were strongly suppressed compared to the response to the single source; this is the condition shown in Figure 5D. Target and masker spike counts diverged markedly as the masker source was shifted away from the target source. In conditions of wide target/masker separation, the response synchronized to the target or masker could be equal in magnitude to the response to the single source. This unit was representative of the majority in the study in that the more contralateral sound source elicited a stronger response than did the more ipsilateral source; there was, however, a sizeable minority of units that favored the more ipsilateral source. Middlebrooks and Bremen (2013) showed that neurons exhibiting a similar preference for contralateral or ipsilateral sound sources tended to form preference-specific ensembles within the cortex.

The right column of panels in Figure 6 shows the sensitivity with which the sounds synchronized to the target and masker could be segregated significantly on the basis of spike counts. In the illustrated example, supra-threshold sensitivity (i.e., *d’* > 1 or < -1) was observed in 5 of the 6 conditions of the masker at the minimum tested separation to the left or right of the target. This unit was representative of the finding that, in most cases, target/masker discrimination was more acute when the target source was located on the midline or in the ipsilateral half of space compared to when the target source was contralateral to the cortical recording site.

The unit in Figures 5 and 6 was representative of essentially all those in the Middlebrooks and Bremen study in that its spatial sensitivity increased markedly when the target was presented with a competing sound source. In Figure 6, for example, the blue and red lines, which represent conditions of competing target and masker sources, demonstrate substantially greater modulation by source location than does the black line, which represents the single-source condition. Across the sampled population, the breadth of tuning in azimuth narrowed by about 1/3 and the depth of modulation by changes in the masker location nearly doubled in the presence of a competing sound (Middlebrooks and Bremen, 2013).

Spatial stream segregation by neurons in the cat’s primary auditory cortex tended to replicate the result that feline psychophysical performance is more acute with high- compared to low-frequency sounds (Javier et al., 2016). Middlebrooks and Bremen (2013) computed a metric of the strength of SSS. That metric varied significantly with the frequency tuning of neurons, indicating that SSS tended to be more robust among neurons tuned to frequencies in the upper half of the range sampled in the cat.

Middlebrooks and Bremen (2013) found that the spike counts synchronized to the target or masker in competing conditions could be modeled well by a linear expression that incorporated the spatial tuning to a single source and the magnitude of the forward suppression (or “attenuation”) that could be measured in the co-localized condition. Forward suppression is a mechanism at one or more levels of the auditory pathway by which the response to one sound suppresses the response to a following sound. Middlebrooks and Bremen confirmed empirically that the forward suppression that they observed was not due to the simple habituation of responses of neurons in the auditory cortex. That observation suggested that forward suppression observed in the cortex is inherited from a sub-cortical level.

The conclusion of a sub-cortical origin of forward suppression is supported by measures of SSS and forward suppression at multiple levels of the rat ascending auditory pathway (Yao et al., 2015). In that study, SSS and forward suppression were essentially absent in the inferior colliculus at stimulus rates at which human and feline psychophysical listeners exhibit spatial stream segregation. Stream segregation and forward suppression first emerged at the level of the nucleus of the brachium of the inferior colliculus. Those phenomena also were robust in about 2/3 of neurons sampled in the ventral nucleus of the medial geniculate and were ubiquitous in the primary auditory cortex. The SSS strengthened at successive levels of the ascending auditory pathway, both due to increasing spatial sensitivity of neurons and increasing forward suppression. Tests of GABA inhibitors applied to the cortical surface demonstrated that forward suppression is not due to synaptic inhibition at the level of the cortex. Instead, Yao and colleagues favored the view that forward suppression underlying stream segregation is most likely due to synaptic depression in the thalamocortical projection.

## Rhythmic Masking Release in the Auditory Cortex

The rhythmic masking release task that was employed in psychophysical experiments in humans (Middlebrooks and Onsan, 2012) and cats (Javier et al., 2016) demonstrated that human and feline listeners could discriminate rhythmic patterns when the target and masker sources were separated by around 10°. That is roughly the spatial acuity with which cortical neurons in the anesthetized cat auditory cortex could segregate streams of noise bursts from alternating source locations, according to the results from Middlebrooks and Bremen (2013). The latter authors extended that observation by testing the target-masker separation at which target rhythm could be identified on the basis of firing patterns of single cortical neurons.

**FIGURE 7:**
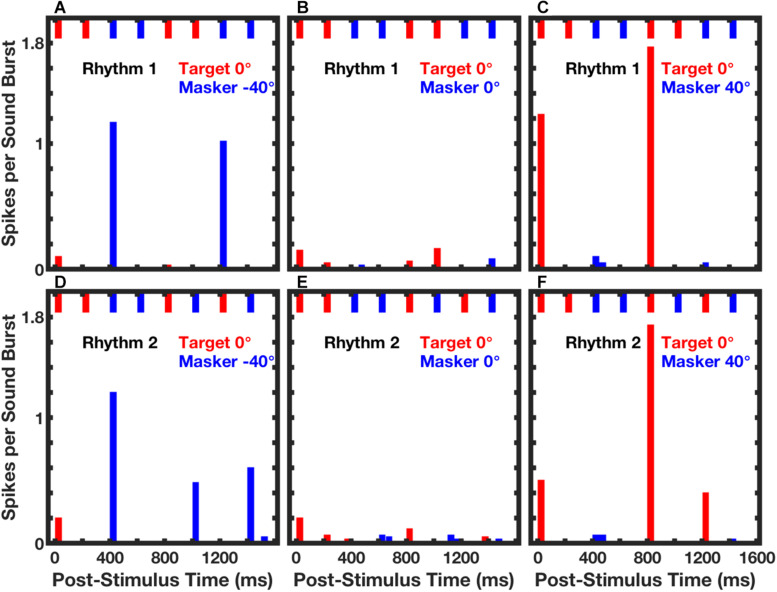
Spike counts from an isolated single neuron synchronized to RMR stimuli. Post-stimulus-time histogram bars show mean spike counts synchronized to noise bursts from the target (red) or masker (blue) source. Data were averaged over 3 continuous repetitions of each rhythm in each of 10 trials. The upper and lower rows of panels represent responses to Rhythm 1 (top) and Rhythm 2 (bottom). The stimulus rhythm is represented by the row of short bars across the top of each panel. Across all panels, the target source was fixed at 0°. The masker source was located at contralateral 40° **(A,D)**, 0° **(B,E)**, or ipsilateral 40° **(C,F)**. Unit 1204.3.11. (From Middlebrooks and Bremen, 2013).

In those empirical tests, stimuli consisted of sequences of broad-band noise bursts presented as Rhythm 1 or Rhythm 2, which were essentially equivalent to the broad-band condition in the human psychophysical experiments (Middlebrooks and Onsan, 2012). The target source was fixed at 0°, and the masker source was varied in azimuth. Neurons synchronized strongly to target or masker components of competing sounds. Figure 7 shows post-stimulus-time histograms from a single well-isolated neuron in response to Rhythm 1 (top row of panels) or Rhythm 2 (bottom row) in three target/masker configurations (columns). The pattern of short bars across the top of each panel represents the stimulus rhythm, consisting in each case of four noise bursts from the target (red) and four from the masker (blue).

The response of that neuron was almost entirely suppressed when the target and masker were co-located (Figures 7B,E). Robust responses synchronized to the target or masker emerged when the masker was shifted to one or the other side of the target source. When the masker source was at contralateral 40° (Figures 7A,D), the neuron responded strongly only to temporally isolated masker bursts. That is, there were strong responses to a masker burst that followed a target burst, but no response to the second of two successive masker bursts. In contrast, when the masker source was at ipsilateral 40°, the response of the neuron was captured by the target sound bursts. In that condition, the response was restricted to target bursts that followed spatially distinct masker bursts, and there was no response to the second of two target bursts.

**FIGURE 8:**
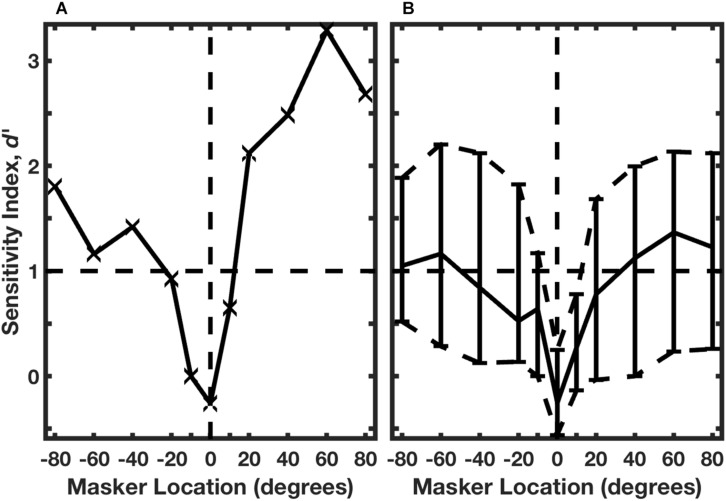
Neural classification of rhythms. **(A)** An isolated single neuron. The target was fixed at 0°, and the masker source was varied parametrically. A regression procedure was used to identify Rhythm 1 or 2 based on the temporal patterns of neural spike counts. Performance is given by *d’*, based on trial-by-trial distributions of spike patterns across 10 trials. The horizontal dashed line indicates the RMR threshold criterion of *d’* = 1. Data are from the same unit represented in Figure 7. **(B)** Distribution of performance across 57 isolated single neurons. The solid curve shows the median, and dashed curves show the 25th and 75th percentiles. (From Middlebrooks and Bremen, 2013).

The identities of the two rhythms are evident by casual inspection of the histograms in Figure 7: there are strong responses at two post-stimulus times in response to Rhythm 1 and at three post-stimulus times in response to Rhythm 2. Middlebrooks and Bremen (2013) used multiple linear regression to evaluate the spike counts in each of 8 time bins (the regressor), solving for the appropriate rhythm, 1 or 2. Figure 8A shows the performance of a single unit in discriminating between stimulus Rhythms 1 and 2; the target was fixed at 0°, and the masker was varied in azimuth. When target and masker were co-located, performance was around chance level. When the masker was shifted to either side, however, performance rapidly improved. Figure 8B shows the distribution of *d’* values for the population of 57 well-isolated units that were tested in the Middlebrooks and Bremen (2013) study; the solid line plots the median, and the dashed lines show the 25^th^ and 75^th^ quartiles. Using a criterion of *d’* = 1, about 25% of neurons segregated streams from target and masker sources separated by as little as about 10°. That acuity of single cortical neurons is remarkably close to the psychophysical thresholds of feline (and human) listeners.

## A Breakdown in Spatial Hearing

The auditory brainstem is well adapted for the fine temporal and intensive processing that is needed for use of interaural difference cues for spatial hearing. These adaptations include the end-bulbs of Held that terminate on the bushy cells of the anterior ventral cochlear nucleus (AVCN), the calyceal endings of Held in the medial nucleus of the trapezoid body (MNTB), and the specialized binaural nuclei of the superior olivary complex, the medial superior olive (MSO) and the lateral superior olive (LSO). All of those structures exhibit a high expression of high-threshold voltage-dependent potassium channels, specifically Kv3.1 and Kv3.3 (Grigg et al., 2000; Li et al., 2001; Chang et al., 2007); Kv3.3 subunits are highly expressed in the AVCN, MNTB, MSO, LSO, and central nucleus of the inferior colliculus (ICc), whereas Kv3.1 is largely restricted to the AVCN, MNTB, and ICc, with relatively little expression in the MSO and LSO (Li et al., 2001). The Kv3.1 and Kv3.3 channels permit rapid repolarization of action potentials, thereby supporting high spike rates and high temporal precision. In the mouse MNTB, either Kv3.1 or Kv3.3 subunits supported rapid repolarization, whereas Kv3.3 was essential for repolarization in the LSO (Choudhury et al., 2020).

Middlebrooks et al. (2013) took advantage of a natural experiment by testing psychophysical performance in human listeners who lack normal function of Kv3.3 channels. Autosomal dominant mutations in the gene encoding Kv3.3 have been identified in two kindreds, one in France (Herman-Bert et al., 2000) and one in the Philippines (Waters et al., 2005; Subramony et al., 2013). Both kindreds exhibit spinocerebellar ataxia 13 (SCA13), although the kindreds differ in channel properties. Study of the mutation in the Filipino kindred, *KCNC3^R420H^*, in frog oocytes has demonstrated dominant negative suppression of potassium conductance (Waters et al., 2006). Middlebrooks et al. (2013) tested the hypothesis that disruption of normal Kv3.3 channel activity would also disrupt sensitivity to interaural difference cues.

Those authors tested 13 affected individuals in the Filipino family as well as control groups consisting of 6 unaffected family members and 16 non-related normal-hearing age-matched individuals. All of the affected participants were shown by molecular testing to be heterozygous for the mutated Kv3.3 gene. The family members were all evaluated for clinical signs of cerebellar ataxia. The clinical status was summarized by the Scale for the Assessment and Rating of Ataxia (SARA). Among the participants carrying the mutated gene, SARA scores ranged from 0 (asymptomatic) to 32.5 (severe disability).

The affected family members showed largely age-appropriate left- and right-ear pure-tone audiograms. None of the family members reported hearing disabilities or hearing-aid use. Dichotic (i.e., binaural) hearing tests utilized low- or high-passed stimuli that were designed to target, respectively, ITD_fs_ and ILD sensitivity and the corresponding pathways. On each trial, listeners heard two sounds and reported whether the second sound was to the left or the right of the first.

**FIGURE 9:**
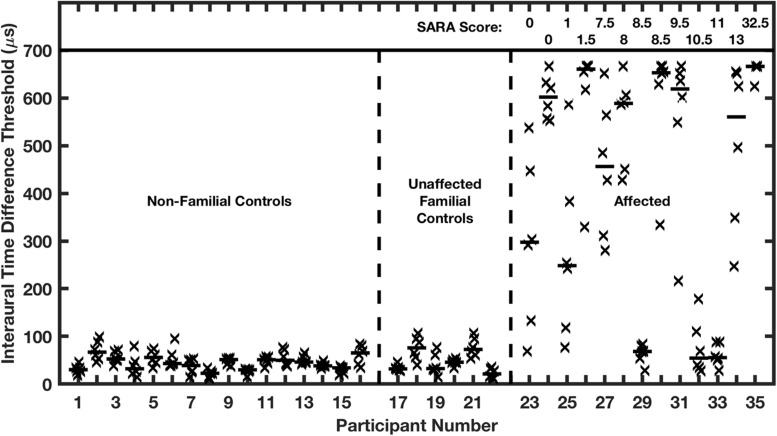
Interaural time difference (ITD) thresholds are elevated in humans affected with a dominant negative mutation in potassium channel Kv3.3. Participants were assigned to 3 groups on the basis of the molecular testing of the *Kv3.3* gene: (1) age-matched non-familial controls; (2) unaffected familial controls; and (3) the affected group. The affected individuals are ranked left to right according to their SARA scores, shown at the top of the figure; SARA is an assessment of ataxia described in the text. Each vertical column of symbols represents 6 threshold measurements (X’s) and a median (horizontal bar) for one participant. Median ITDs in the two control groups were not significantly different from each other and were comparable to published reports of ITDs of untrained listeners. The median ITDs in the affected group were significantly greater than those in the control groups. (From Middlebrooks et al., 2013).

Nearly all the affected family members exhibited marked elevations of ITD and ILD thresholds. In the case of ITD (Figure 9), control groups showed median thresholds around 45 μs, which is comparable to published thresholds of untrained normal-hearing listeners (Wright and Fitzgerald, 2001). Conversely, ten of the 13 affected participants had ITD thresholds significantly higher than the thresholds of any of those in the control groups, mostly higher than 500 μs, which is near the maximum value produced by free-field sounds. The remaining 3 affected participants had median ITD thresholds of 68, 55, and 56 μs, which are within the distribution of control medians. Remarkably, there was no systematic correlation in the affected group between ITD thresholds and ataxia, as represented by SARA scores. Thresholds higher than 500 μs were exhibited by participants having the lowest (i.e., best: 0) or highest (32.5) SARA scores in the sample, and participants having SARA scores higher than 8 had ITD thresholds ranging from <100 to >650 μs. It is worth noting that SARA scores are based on fairly rudimentary motor exams, such that a score of 0 sometimes will be assigned in a case in which later, more precise, measures might reveal a gait disturbance or other signs of ataxia.

**FIGURE 10:**
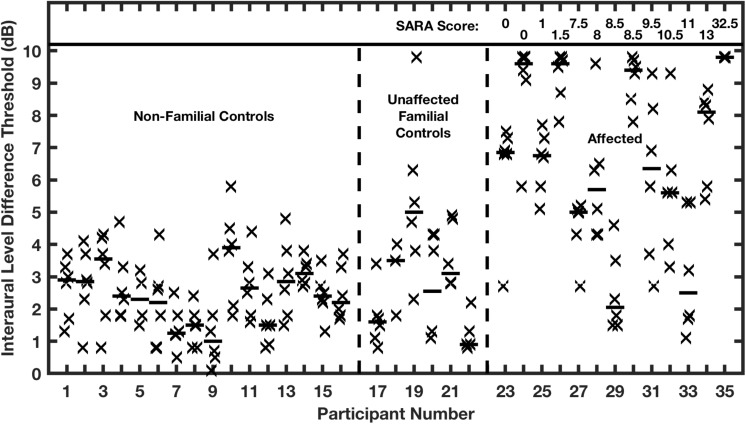
Interaural level difference (ILD) thresholds are elevated in humans affected with a dominant negative mutation in potassium channel Kv3.3. ILD thresholds are shown for two control groups and the affected group. Other conventions are as in Figure 9. (From Middlebrooks et al., 2013).

Thresholds for ILD detection were similarly elevated (Figure 10). All but 2 of the affected individuals had ILD thresholds that were 5 dB or greater, in contrast with the control groups having median values that all were 5 dB or less, averaging 2.5 dB. Again, there was no correlation in the affected group between ILD threshold and ataxia. Within the affected group, ITD thresholds of affected individuals correlated highly with their ILD thresholds. The high correlation between deficits in ITD and ILD sensitivity, and the absence of correlation with the severity of ataxia, suggests that expression of the mutant allele and selection of channel subtypes might differ between auditory and cerebellar pathways. Moreover, the presence of functional Kv3.3 subunits within voltage-gated potassium channels might be more or less essential for rapid repolarization in various structures, as has been demonstrated in the mouse LSO and MNTB (Choudhury et al., 2020).

It was not feasible for Middlebrooks et al. (2013) to test SSS in the affected listeners. Nevertheless, one could speculate that the deficits in ITD_fs_ and ILD sensitivity would severely impair SSS, leading to great difficulty in parsing conversations in the presence of competing sounds. In principle, spectral-shape cues could replace binaural cues to provide spatial information in the horizontal dimension. Tests of horizontal sound-source localization in the absence of binaural cues, however, have yielded rather mixed results (Belendiuk and Butler, 1977; Slattery and Middlebrooks, 1994; Wightman and Kistler, 1997; Macpherson and Middlebrooks, 2002) and we are aware of no tests of SSS in the horizontal dimension have evaluated the contribution of spectral-shape cues. The tests of SSS by Middlebrooks and Onsan (2012) in the horizontal plane and in the vertical midline suggest that the most robust, highest-acuity, SSS relies on binaural cues, specifically ITD_fs_ and ILD.

## Summary and Conclusion

How does a listener piece string together the syllables from one talker amid the tangle of competing voices at a cocktail party or any other complex auditory scene? What are the brain mechanisms that enable such a task? In this review, we have focused on a series of experiments that were unified by use of a common psychophysical task, “rhythmic masking release”, and its corresponding stimulus set. Here, we summarize some of the key conclusions from those previous studies.

In psychophysical tests, listeners successfully segregated interleaved sound sequences that differed only in the locations of target and masker. This suggests that spatial hearing would be highly beneficial in isolating a single talker amid other competing sounds. Among potential acoustical spatial cues, the best psychophysical SSS performance was provided by interaural difference (binaural) cues, particularly ITD_fs_ in humans. Nevertheless, SSS was possible for locations in the vertical midline, where interaural cues are negligible. This indicates that SSS is not strictly a binaural phenomenon.

Elementary acoustical cues for spatial hearing are analyzed in specialized nuclei of the auditory brainstem. The high-voltage voltage-gated potassium channel, Kv3.3, is particularly important for brainstem processing of ITD_fs_ and ILD. In a human kindred bearing a dominant negative mutation in the gene for the Kv3.3 channel, affected individuals showed a lack of sensitivity for ITD_fs_ and ILD, which almost certainly would severely impair their use of spatial hearing in everyday complex listening situations. Physiological studies in animal models demonstrate that SSS is derived from spatial and forward-suppression mechanism in the auditory brainstem, emerging in full force in the thalamo-cortical projection. Single neurons in the primary auditory cortex of the cat exhibit SSS with spatial acuity comparable to psychophysical listeners. The observation that SSS is observed in an early cortical level in the presence of anesthesia, i.e., in the absence of higher-level cortical processes, further supports the view that brainstem and thalamocortical mechanisms have already done the work of sorting interleaved sequences of sounds into activity in multiple distinct populations of cortical neurons.

In the cat auditory cortex, neurons that synchronize preferentially to the leftmost of a pair of sound sources tend to cluster apart from those that synchronize to the rightmost source. To the degree that the cat results can be generalized to humans, the single-neuron results provide a picture of what might be going on in our cocktail-party listener’s brain when he or she attempts to focus on a speech stream from a particular talker. We speculate that the speech stream of interest would activate one or more ensembles of mutually synchronized neurons that would be distinct from ensembles synchronized to other speech streams, background music, clinking glasses, etc. The listener, then, could use higher-level auditory or pre-frontal mechanisms to shine a light on the neural ensemble(s) representing the talker of interest. One hopes that this view of active cortical mechanisms can be tested, with or without a cocktail in hand, in future studies in behaving animals.

## Author Contributions

JM wrote the manuscript with comments and suggestions from MW. Both authors contributed to the article and approved the submitted version.

## Conflict of Interest

The authors declare that the research was conducted in the absence of any commercial or financial relationships that could be construed as a potential conflict of interest.
